# Occurrence of Pre- and Post-Harvest Mycotoxins and Other Secondary Metabolites in Danish Maize Silage

**DOI:** 10.3390/toxins6082256

**Published:** 2014-07-31

**Authors:** Ida M. L. Drejer Storm, Rie Romme Rasmussen, Peter Have Rasmussen

**Affiliations:** 1 Center for Microbial Biotechnology, Department of Systems Biology, Technical University of Denmark, Building 221, DK-2800 Kgs. Lyngby, Denmark; 2 Department of Food Chemistry, National Food Institute, Technical University of Denmark, Mørkhøj Bygade 19, DK-2860 Søborg, Denmark; E-Mail: riro@food.dtu.dk

**Keywords:** maize, silage, mycotoxins, secondary metabolites, occurrence, cattle feed, multi mycotoxin, LC-MS/MS

## Abstract

Maize silage is a widely used feed product for cattle worldwide, which may be contaminated with mycotoxins, pre- and post-harvest. This concerns both farmers and consumers. To assess the exposure of Danish cattle to mycotoxins from maize silage, 99 samples of whole-crop maize (ensiled and un-ensiled) were analyzed for their contents of 27 mycotoxins and other secondary fungal metabolites by liquid chromatography-tandem mass spectrometry. The method specifically targets the majority of common pre- and post-harvest fungi associated with maize silage in Denmark. Sixty-one samples contained one or more of the 27 analytes in detectable concentrations. The most common mycotoxins were zearalenone, enniatin B nivalenol and andrastin A, found in 34%, 28%, 16% and 15% of the samples, respectively. None of the samples contained mycotoxins above the EU recommended maximum concentrations for *Fusarium* toxins in cereal-based roughage. Thus, the present study does not indicate that Danish maize silage in general is a cause of acute single mycotoxin intoxications in cattle. However, 31 of the samples contained multiple analytes; two samples as much as seven different fungal metabolites. Feed rations with maize silage may therefore contain complex mixtures of fungal secondary metabolites with unknown biological activity. This emphasizes the need for a thorough examination of the effects of chronic exposure and possible synergistic effects.

## 1. Introduction

Contamination of animal feed with mycotoxins is of concern for both farmers and consumers of animal products. Maize silage is a widely used feed product for cattle around the world, particularly in dairy production [1]. It is used year round, and a dairy cow may consume 25 kg dry matter per day [2]. Maize silage may be contaminated with various fungal metabolites both pre- and post-harvest. Common pre-harvest contaminants are species of *Fusarium*, *Alternaria* and *Aspergillus*, while post-harvest infection is most often caused by *Penicillium roqueforti*, *P. paneum*, Zygomycetes, *Aspergillus fumigatus*, *Byssochlamys nivea* and a few other fungi [3,4].

Mycotoxin contamination caused by fungi can affect animal health [5] and productivity [6]. The general symptoms of mycotoxicosis include loss of appetite, poor weight gain, feed refusal, diarrhea, bleeding, birth defects and kidney, liver or lung damage [7]. Acute intoxications of animals are rare [8], but it is important to know the exposure of animals, since a chronic exposure to low levels of mycotoxins can give non-specific symptoms, such as impaired immune system and increased infections or metabolic and hormonal imbalances [6,9]. Moreover, little is known about the possible synergistic effects of mycotoxins, and the diagnosis of mycotoxicosis can be difficult, because other diseases may give similar symptoms [6].

The fungi spoiling maize and maize silage are able to produce a wide range of secondary metabolites on different substrates [10]. Previous studies of mycotoxins in maize silage and whole-crop maize for silage have detected various fungal metabolites of pre- and post-harvest origin [11,12,13,14,15,16,17,18,19,20,21,22,23]. The study by [11] was the most comprehensive on maize silage, covering 140 samples from the Netherlands, which were analyzed for 20 different mycotoxins, including aflatoxins, deoxynivalenol, zearalenone and ochratoxin A, but only a few compounds produced by common post-harvest silage contaminants. This study showed that the *Fusarium* toxins, deoxynivalenol and zearalenone, were commonly present at levels below the maximum recommended concentrations. Mycophenolic acid and roquefortine C produced by the post-harvest silage contaminant, *P. roqueforti*, were analyzed, but not detected. This may be because the silage samples were taken in October and November, where maize silages are only a few weeks old, thus reducing the possibility of encountering post-harvest toxins. However, maize silage can also contain high levels of post-harvest fungal metabolites in areas with visible fungal growth [18], whose presence is only sparsely examined and not regulated.

The carry-over of mycotoxins and their metabolites to edible animal products, such as milk and meat, is a potential risk for consumers. Aflatoxins have been most extensively regulated, but also: the trichothecenes, deoxynivalenol, diacetoxyscirpenol, T-2 toxin and HT-2 toxin; the fumonisins B1, B2 and B3; the ergot alkaloids; and ochratoxin A and zearalenone have been regulated in feed by some countries [24]. In the EU, maize roughage intended for animal feed is recommended not to contain more than 2 mg/kg zearalenone, 8 mg/kg deoxynivalenol and 60 mg/kg fumonisins (sum of B1 and B2) [25]. For maize intended for silage grown in Northern Europe, the risk of aflatoxins and fumonisins is, however, very little, as both groups of mycotoxins are produced by fungi that require arid, semi-arid, sub-tropical or tropical climate conditions [26,27]. The absence of aflatoxin producing fungi in Danish maize silage was confirmed by [28], where only one single isolate of *A. flavus* was obtained during a long-term survey of silage microbiota in 20 Danish silage stacks. Similarly, [11] did not detect aflatoxin B1 in any samples of maize, grass and wheat silages from the Netherlands. The very low risk of fumonisins in Danish maize for silage was confirmed in a four-year survey of *Fusarium* toxins in maize conducted from 2004–2007 on a total of 239 samples. Summarizing the results, [27] shows that the average concentrations of fumonisins B1 and B2 all four years were below 0.10 mg/kg, and the maximum observed was 2.27 mg/kg of fumonisin B1.

With the selected multi-mycotoxin method, we are capable of determining 27 mycotoxins and other fungal secondary metabolites in maize silage samples [18] of relevance for present North European climate conditions. It is specifically developed and validated for maize silage and detects metabolites from most of the common fungal contaminants of silage, both pre- and post-harvest [4]. It is therefore uniquely able to give an estimate of the overall exposure to mycotoxins through maize silage. This study covers 99 samples of maize silage and whole-crop maize for silage analyzed for alternariol (AOH), alternariol monomethyl ether (AME), altersetin (ALS), cyclopiazonic acid (CPA), deoxynivalenol (DON), enniatin B (ENN B), nivalenol (NIV), sterigmatocystin (STE), T-2 toxin (T-2), tenuazonic acid (TEA) and zearalenone (ZEA), all associated with the field mycobiota, and andrastin A (AND A), citreoisocoumarine (CICO), fumigaclavine A (FUC A), fumigaclavine C (FUC C), fumitremorgin A (FUT A), gliotoxin (GLI), marcfortine A (MAC A), marcfortine B (MAC B), mevinolin (MEV), mycophenolic acid (MPA), ochratoxin A (OTA), patulin (PAT), penitrem A (PEN A), PR toxin (PR), roquefortine A (ROQ A) and roquefortine C (ROQ C) produced by storage fungi.

## 2. Results and Discussion

### 2.1. Method Performance

Initially in the data analysis, a range of method performance parameters were measured and compared to the measurements from the method validation conducted previously [18]. The LODs of the method as determined during validation are presented in Table 1, together with the values for the limits of quantification (LOQ). Co-eluting matrix compounds early in the chromatogram did interfere with the most polar analytes (PAT, NIV and DON), which resulted in high LOD values for these analytes (0.37, 0.12 and 0.74 mg/kg, respectively). However, for DON, the interference was negligible at concentrations near or above the guideline value for maximum content in animal feed (8 mg/kg). No such guideline values exist for PAT and NIV in current EU legislation [25,29].

The method recoveries were determined for DON, GLI, NIV, PAT, ROQ C and T-2 in spiked samples (*n* = 6) resulting in average recoveries of 91%, 79%, 67%, 93%, 110% and 114%, respectively. The recoveries for DON, GLI, NIV and PAT were in good agreement with the recoveries determined during method validation [18]. For ROQ C and T-2, the recoveries of 110% and 114% were, by themselves, acceptable, but not comparable to the previous validation results of 205% and 55%, respectively. The current sample clean-up was performed with more experience and analyzed in shorter series for this study than during the original validation, which may be the reason for the better average recoveries.

**Table 1:** Mycotoxins and other secondary fungal metabolites included in the present study, their abbreviations and limits of detection (LOD) and quantification (LOQ) for the quantitatively determined analytes as determined during method validation [18].

	Analyte	Abbreviation	Mean	Reproducibility	LOD (µg·kg^−1^)	LOQ (µg·kg^−1^)
			Recovery (%)	*RSD*_IR_ (%)		
Quantitative	Alternariol	AOH	78	14	10	20
	Alternariol momomethyl ether	AME	79	10	6	12
	Andrastin A	AND A	122	12	1	2
	Cyclopiazonic acid	CPA	63	35	15	30
	Deoxynivalenol	DON	83	18	739	1478
	Enniatin B	ENN B	60	24	24	48
	Fumitremorgin A	FUT A	93	23	76	152
	Gliotoxin	GLI	85	13	71	142
	Mevinolin	MEV	68	27	25	50
	Mycophenolic acid	MPA	90	13	7	14
	Nivalenol	NIV	68	15	122	244
	Ochratoxin A	OTA	71	9	10	20
	Patulin	PAT	100	17	371	742
	Penitrem A	PEN A	107	12	8	16
	Roquefortine C	ROQ C	205	25	158	316
	Sterigmatocystin	STE	72	9	8	16
	T-2 toxin	T-2	55	26	96	192
	Tenuazonic acid	TEA	37	20	121	242
	Zearalenone	ZEA	90	16	9	18
Qualitative	Altersetin	ALS	91	14	-	-
	Citreoisocoumarin	CICO	84	7	-	-
	Fumigaclavine A	FUC A	93	21	-	-
	Fumigaclavine C	FUC C	176	13	-	-
	Marcfortine A	MAC A	63	16	-	-
	Marcfortine B	MAC B	61	9	-	-
	PR-toxin	PR	56	32	-	-
	Roquefortine A	ROQ A	103	32	-	-

Recoveries of ROQ C from fresh extracts of a spiked sample were on average 110% (*n* = 6), while recoveries of extract stored at −20 °C for 1–3 months were on average 62% (*n* = 6). This difference in recoveries was significant (*p* < 0.001). This drop in recoveries of ROQ C indicates a degradation of ROQ C in extracts during storage. A maximum storage time for sample extracts of three days at −20 °C before analysis was therefore implemented.

The relative standard deviation (*RSD*_IR_) was also determined for DON, GLI, NIV, PAT, ROQ C and T-2 in the spiked samples (*n* = 6), resulting in values of 21%, 26%, 19%, 11%, 15% and 32%, respectively. These values were all comparable to RSD_IR_ obtained in the original method validation [18].

### 2.2. Mycotoxins in Maize and Maize Silage

Out of the 99 analyzed samples, 61 contained one or more of the detectable analytes in concentrations above LOD. Summary statistics for the findings of each of the analytes are presented in Table 2, and a list of all positive results is available as Supplementary Information (Table S1).

**Table 2:** Summary statistics on the contents of mycotoxins detected in fresh, whole-crop maize samples, ensiled maize samples and all 99 samples together. For compound abbreviations, see Table 1. The number of samples with concentrations above LOD (*n*_pos_) are included for both quantitatively and qualitatively determined compounds. For quantitatively determined compounds, the average concentration of positive samples (*avg*_pos_) with the standard error of the mean (SEM) in parentheses and the maximum concentrations (max) are presented in µg/kg fresh weight.

		Fresh Maize (*n* = 17)			Ensiled Maize (*n* = 82)			Total (*n* = 99)		
	Compound	*n*_pos_	*avg*_pos_ (SEM)	Max	*n*_pos_	*avg*_pos_ (SEM)	Max	*n*_pos_	*avg*_pos_ (SEM)	Max
Quantitative	AME	1	11	11	2	8(1)	8.8	3	9(1)	11
	AND A				15	169(54)	691	15	169(54)	691
	AOH				2	18(6)	24	2	18(6)	24
	DON	2	2369(293)	2,662	5	1629(365)	2,974	7	1841(293)	2974
	ENN B	8	128(40) ^a^	365	20	53(7) ^b^	152	28	75(13)	365
	MPA				2	43(9)	52	2	43(9)	52
	NIV	5	255(37)	351	11	266(53)	758	16	263(38)	758
	ROQ C				2	173(15)	189	2	173(15)	189
	ZEA	11	83(59)	666	23	66(15)	311	34	71(21)	666
Qualitative	CICO	1			7			8		
	MAC A				6			6		
	MAC B				1			1		
	ROQ A				9			9		

^a,b^ Group means with different superscript letters differ significantly from each other (*p* ≤ 0.05).

#### 2.2.1. *Fusarium* Toxins

The most common mycotoxins in the 99 samples were the *Fusarium* metabolites, with ZEA, ENN B and NIV being detected in 34%, 28% and 16% of the samples, respectively. This is consistent with previous surveys showing a widespread growth of *Fusarium* in North European maize plants [11,21,22]. The concentrations of the *Fusarium* toxins ZEA, DON and ENN B detected in the present study were similar to results from the studies by [11] and [22]. However, for ZEA and NIV, the concentrations and occurrences were higher in the German study by [21], where the small amount of maize and maize silage samples originated from the southern part of Germany. Similarly, the frequency of DON observed by [11] was higher than in the present study. This difference in frequency can be attributed to a difference in LOD. The method used for the present study focused on a wide selection of metabolites rather than low LODs, resulting in a lower number of positive samples, but still capturing all samples with concentrations near regulatory levels.

None of the analyzed samples contained DON or ZEA in concentrations above the guidance values set for individual feeding products by the European Commission [25]. Fusarium mycotoxins in maize silage are therefore not likely to be the cause of the general occurrence of acute intoxications of Danish cattle. However, three samples contained DON and three samples ZEA in values above the guidance values for complete feedstuffs to dairy cattle, which are 5000 and 500 µg/kg, respectively. Two of these samples (#9 and #99) were the same, thus having high levels of both DON and ZEA. DON and ZEA are known to have immunosuppressive effects and estrogenic effects, respectively [30,31]. With silage constituting up to 50%–75% of the daily feed ration to dairy cattle [12], such concentrations may affect the animals.

DON is also part of the trichothecene group, which comprises numerous fungal metabolites, of which, e.g., NIV, scirpentriol, 15-monoacetoxyscirpenol, HT-2 toxin, T-2 and diacetoxyscirpenol (DAS) have been associated with maize and silage [21]. NIV was detected in 16% of samples, but the risk for animals and public health caused by NIV in animal feed remains unassessed [32]. However, the T-2 concentration and occurrence was low, and DAS was not determined in the present study. For the enniatins, *in vitro* data suggest biological activity; however, there is a clear lack of animal studies, and more data is needed to evaluate their toxicity [33].

#### 2.2.2. *Penicillium* Toxins

The second most common group of secondary fungal metabolites was composed of the post-harvest metabolites, AND A, ROQ C, MAC A and CICO. with AND A being the most common (15% of samples). They are all produced by *P. roqueforti* or *P. paneum* [34]. The metabolite abundance of and instrument sensitivity for AND A makes it a good marker for the presence of these species in silage. The low occurrence of the *P. roqueforti*/*P. paneum* metabolites, MPA and ROQ C, were in line with [11], who did not detect these toxins in 140 maize silages sampled from sealed stacks, but lower than in a similar study with samples taken from the cutting front of silage silos [12]. *Penicillium roqueforti* and *P. paneum* have been associated with ill-thrift and disease in cattle herds [4]. However, no direct effects were observed at high doses of MPA and ROQ C in two sheep studies [35,36] and no adverse effects have been described for AND A [37]. It therefore remains un-answered whether the presence of these *P. roqueforti*/*P. paneum* metabolites in silage poses a health and production problem for dairy cattle.

#### 2.2.3. *Alternaria* Toxins

*Alternaria* toxins are produced pre-harvest in maize [15], but the presence of AOH and AME in maize silage is only recently described [18]. Their occurrence and concentrations in the present study were low. Seven samples contained at least traces of these analytes, and the co-occurrence of these compounds is a good marker for pre-harvest infection with *Alternaria*. The toxicity of alternariols is not well examined [38]. *In vitro* experiments show that alternariols have DNA strand-breaking activities [39]. *Alternaria* toxins have also been associated with human esophageal cancer in China [40]. It is therefore important to be aware of the possibility of *Alternaria* toxins in silage, but possible effects on animals or carry-over to products for human consumption is not sufficiently examined.

#### 2.2.4. Other Fungal Metabolites

Other fungal metabolites were marked by their absence, rather than presence, for instance none of the secondary metabolites from *Aspergillus fumigatus* (GLI, FUT A, FUC A, FUC C) were detected in the present study. *A. fumigatus* is commonly isolated from silages in both warm and temperate climates [4], including Danish maize silage [28]. It produces gliotoxin, which has been detected in silage by [18,19,41]. The absence in this survey therefore indicates that the mycotoxin production of this fungus is limited under Danish conditions, even though the fungus is generally present. PAT and CPA were also not detected in the present study. The high occurrence of PAT, CPA, MPA and ROQ C observed by [14] could indicate climatic/continental differences or poor silage management, but the risk of false positive results in that study must also be considered high, because of the non-selective LC-MS method applied and because the recovery was tested high above the LOD. Absence of CPA was expected, as *A. flavus* is mainly a problem in warmer climates than the Danish one [26]. Likewise, the producers of aflatoxin B1 are not relevant under Danish climatic conditions [26,38], and aflatoxin B1 was therefore not included in the applied detection method. The same was the case for fumonisin B1 and B2, due to the low levels of these mycotoxins detected in 239 Danish maize samples from 2004–2007 [27].

#### 2.2.5. Multiple Mycotoxins in the Same Samples

**Figure 1:**
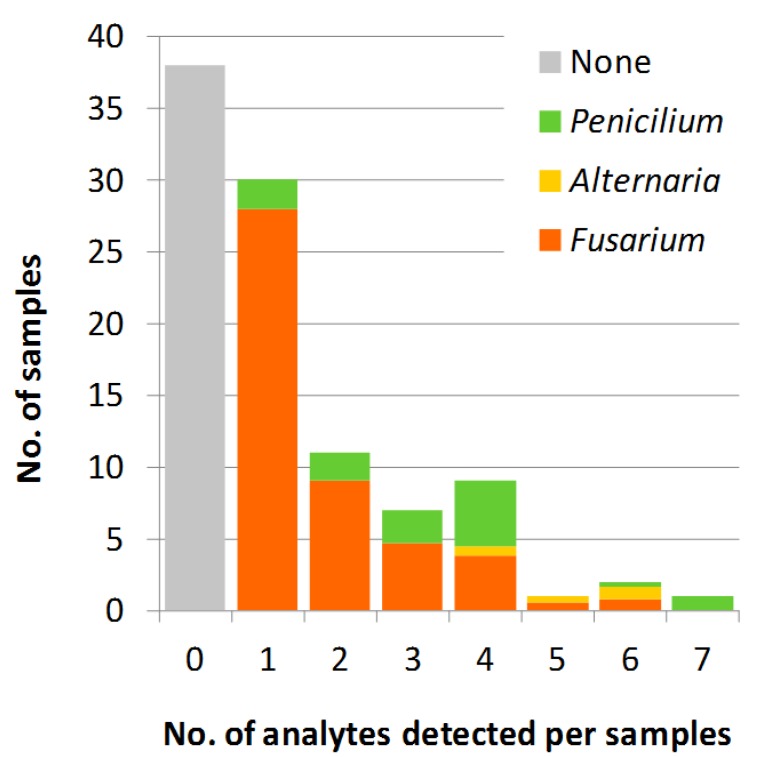
Distribution of the 99 maize silage samples according to the number of analytes detected in each sample. The frequency of the fungal species in the samples is illustrated by colors relative to the total number of infections in the sample category.

Thirty-one of the total of 99 analyzed samples contained more than one analyte, with two samples containing as much as seven analytes (Figure 1). Sample #9 contained the following toxins (concentrations (µg/kg) in brackets where applicable): AME (8.8), AOH (12), ALS, DON (2974), ENN B (85), NIV (758) and ZEA (209); thus showing infection with both the *Fusarium* and *Alternaria* pre-harvest species. Sample #27 contained AND A (521), CICO, MAC A, MAC B, MPA (34), ROQ A and ROQ C (158), all known to be produced by the common post-harvest species, *P. roqueforti* and *P. paneum*.

The finding of approximately one third of samples being infected with multiple secondary metabolites raises the issue of possible synergistic effects during multiple exposures. Alongside with this comes the question of the possible effects of long-term exposure to low concentrations of secondary metabolites. The majority of the silage samples in this study were taken approximately six months after ensiling, and they were therefore likely to represent the largest selection of post-harvest metabolites expected during the year, as maize silage has been shown to contain the highest amounts of fungal propagules 5–7 months after ensilage [28]. Several of the mycotoxins detected in Danish maize silage are known to have immunosuppressive effects. Besides the trichothecenes, DON and NIV, it includes GLI and MPA at high doses [30,37,42]. The general toxicity and immunotoxicity are considered to be the most critical effects of several trichothecenes [43]. The European Food Safety Authority [44] states the main effects of long-term dietary exposure of animals to DON as weight gain suppression, anorexia and altered nutritional efficiency, but continuous exposure to low levels of immunosuppressive toxins may increase an animal’s susceptibility to infectious diseases [45]. Long-term exposure to multiple mycotoxins, as seen in Sample 9 and 27, may thus result in unknown effects. Unfortunately, long-term *in vivo* studies evaluating the immunosuppressive effects of mycotoxins are sparse [12]. Similarly, very little is known about the *in vivo* toxicological effects of multiple mycotoxins, except for the trichothecenes [46], and the possible synergistic effects of such mixtures should therefore be examined.

#### 2.2.6. Sample Origin and Storage Effects

The findings of mycotoxins in fresh whole-crop maize samples collected prior to ensiling *vs.* the findings in ensiled maize are summarized in Table 2. All of the detected toxins were observed in ensiled maize, while the fresh samples only contained the pre-harvest toxins, ENN B, ZEA, NIV, DON and AME.

The occurrence of pre- and post-harvest mycotoxins was, in general, consistent with the sample origin: maize samples only contained pre-harvest metabolites, while maize silage samples contained both pre- and post-harvest metabolites. The exception was one single finding of CICO in a fresh maize sample. This may be explained by the presence of *P. roqueforti*/*P. paneum*, also prior to ensiling, as shown by [47] or originate from other fungi, e.g., *Phoma* [27].

In accordance with previous studies, the results also indicate that some degradation or transformation of pre-harvest metabolites occurs during ensiling. For DON, ENN B and ZEA, the concentrations, as well as the percentages of positive samples were higher in the fresh maize samples than the ensiled, but only the average concentrations of ENN B differed significantly (*p* < 0.05). Similarly, [21] found a higher abundance and higher concentrations of T-2, HT-2, T-2-tetraol and T-2-triol in maize plants than in maize silage. For the interpretation of this result, it must be taken into account that the statistical analysis in this part of the study is a comparison of average values of two independent samples of, on the one hand, freshly harvested maize for ensiling and, on the other, maize silage. It was not conducted or analyzed as a stability study with an analysis of paired samples before and after ensiling. Further stability studies could involve samples from the same maize being analyzed before and after ensiling.

The sampling procedures used in this study may also have had an effect on the results, due to the inhomogeneous distribution of toxins in the samples taken in fresh and ensilaged maize. Representative sampling of large immobile stacks is always problematic and, for silage, further complicated by the fact that drilling sample holes in the stack may harm the future quality of the silage. It can also be discussed whether silage samples should be taken by drilling or from the cutting face of the silo or stack. While drilling multiple full-depth holes and combining all samples to a composite sample will give a sample representing the entire stack, it poses a lot of work and may harm the conservation of the silage. Taking multiple samples from the cutting face only samples a small part of the stack, but is highly representative for what is being fed to animals at the time of sampling. To achieve results representative of the whole stack by sampling from the cutting face, it will therefore be necessary to do repeated sampling with a relevant time interval. This will, however, give a better impression of the exposure of livestock to mycotoxins.

The samples were collected from 2007 to 2009, thus representing maize grown in 2006, 2007 and 2008. Due to the imbalanced distribution of sample types on sample year and the limited amount of samples with quantifiable concentrations of analytes, it was not possible to conduct a statistical comparison of mycotoxin occurrence between years in this study.

## 3. Experimental Section

### 3.1. Sample Collection and Preparation

Ninety-nine samples of maize silage (*n* = 82) or freshly harvested maize (*n* = 17) intended for silage were gathered. Of the ensiled maize samples 74% were collected when silages were approximately 6 months old. The samples were compiled from different studies conducted over the whole of Denmark from 2007 to 2009, thus incorporating maize from the growth seasons 2006, 2007 and 2008. Samples #1–21 were collected by the Danish Plant Directorate from randomly selected farms. Approximately ten grab samples were collected from the cutting face of the silage stack or silo to form a composite sample. Samples #22–82 were silage samples collected at randomly selected dairy farms in Jutland. Twenty of these samples were collected in 2007 [48] and 41 in 2009 [49]. All of the Samples #22–82 were collected from the full depth of silage stacks with a silage drill approximately 1 m behind the cutting face of the silage stack. Samples #83–99 were field samples of whole fresh maize plants taken at the field level from all over Denmark and consisted of different maize cultivars. The samples were harvested in October, 2007 and 2008, by personnel from the Danish Agricultural Advisory Service, either by hand or by forage harvester.

Samples were homogenized and comminuted by two different methods. Samples #22–62 and #81–97 were freeze dried and milled. From all other samples, a portion of approximately 150 g was frozen by pouring liquid nitrogen over it. As soon as the nitrogen was evaporated, the samples were homogenized in a small domestic blender to a fine powder. All samples were stored at −20 °C until extraction and analysis.

### 3.2. Extraction

A fast and simple pH-buffered extraction was performed according to [18]. The method employs extraction with acetonitrile and water combined with phase-separation induced by the addition of MgSO_4_, a principle known as QuEChERS (Quick, Easy, Cheap, Effective, Rugged, Safe) [50]. The method was developed for non-dried silage samples with a dry matter (DM) content of approximately 0.35 kg DM/kg, of which, 10.0 g of fresh weight silage is used for extraction. A minor modification was included for the analysis of freeze dried samples in the present study, where 3.5 g of dried sample was used together with 6.5 mL of water, thus totaling 10.0 g. The water/acetonitrile ratio in the final sample extraction was therefore approximately the same in the procedures for freeze dried and non-dried silage samples. The use of a relatively large portion of sample for extraction is important to account for the difficulty of homogenizing fresh maize and silage samples.

### 3.3. Sample Analysis

The extracts were analyzed by liquid chromatography- tandem mass spectrometry (LC-MS/MS), as described by [18], with the limits of detection (LOD) presented in Table 1 together with the limits of quantification (LOQ). The values for LOD and LOQ were determined as three- and six-times the standard deviation at intra-laboratory conditions (*SD*_IR_) divided by the recovery, both based on results from the lowest accepted spike levels. Note that eight compounds were determined qualitatively due to a lack of quantitative standards.

The 99 samples were analyzed in 6 separate series on separate days. Each series included 15–20 silage sample extracts, a 6-level matrix matched standard curve of the quantitatively available standards and 1-level of the qualitatively available standards in a matrix matched solution. To compare the performance of each series to previous validation results, one blank silage sample spiked before extraction with 6 mycotoxins (DON, NIV, GLI, PAT, ROQ C and T-2) was included in each series.

### 3.4. Data Analysis

All results are reported without correction for recovery. For comparison with guidance values, a dry matter content of 0.35 kg DM/kg silage was applied.

All analytical series were compared to the validation results for the method with regards to recovery and relative standard deviation under intra-laboratory reproducibility conditions (*RSD*_IR_) for the spiked samples. The *RSD*_IR_ was also calculated on the basis of the results from the naturally-contaminated control sample in each series.

A comparison of mycotoxin concentrations in different sample types was conducted by a homoscedastic two-tailed Students *t*-test in SAS (v 9.1.3, SAS Institute Inc., Cary, NC, USA). A significance level of *p* ≤ 0.05 was applied.

## 4. Conclusions

On the basis of the present study, it is unlikely that Danish maize silage could be the direct cause of acute intoxications in dairy cattle. None of the regulated toxins were detected in concentrations above the guideline values recommended by the European Commission. This does, however, not exclude the possibility of occasional incidences of high contamination levels. The present study also shows that contamination with low levels of multiple secondary metabolites is common. Feed rations with maize silage may therefore contain complex mixtures of fungal secondary metabolites with unknown biological activity. This risk is particularly pronounced in ensiled maize samples, which can contain both pre- and post-harvests metabolites. The possible synergistic effects and effects of long-term exposure to such mixtures are not known, and further research on this subject is recommended.

## Supplementary Files

Supplementary File 1

## Abbreviations

ALS: altersetin
AME: alternariol monomethyl ether
AND A: andrastin A
AOH: alternariol
CICO: citreoisocoumarine
CPA: cyclopiazonic acid
DAS: diacetoxyscirpenol
DON: deoxynivalenol
ENN B: enniatin B
FUC A: fumigaclavine A
FUC C: fumigaclavine C
FUT A: fumitremorgin A
GLI: gliotoxin
LC-MS/MS: liquid chromatography-tandem mass spectrometry
LOD: limit of detection
LOQ: limit of quantification
MAC A: marcfortine A
MAC B: marcfortine B
MEV: mevinolin
MPA: mycophenolic acid
NIV: nivalenol
OTA: ochratoxin A
PAT: patulin
PEN A: penitrem A
PR: PR toxin
QuEChERS: Quick, Easy, Cheap, Effective, Rugged, Safe, a multi-method developed for the analysis of pesticide residues in fruit and vegetables
ROQ A: roquefortine A
ROQ C: roquefortine C
RSD: relative standard deviation
RSD_IR_: the relative standard deviation under intra-laboratory reproducibility conditions
SD_IR_: standard deviation at intra-laboratory conditions
STE: sterigmatocystin
T-2: T-2 toxin
TEA: tenuazonic acid
ZEA: zearalenone
