# Supplementary material for: Occurrence of Pre- and Post-Harvest Mycotoxins and Other Secondary Metabolites in Danish Maize Silage
Source: Toxins (Basel). 2014 Jul 31;6(8):2256–69. doi: 10.3390/toxins6082256 (PMC4147581; doi:10.3390/toxins6082256)
Supplement: Supplementary File 1 [file toxins-06-02256-s001.pdf]

Supplementary Information

Tabel S1. Table of all samples with concentrations of the analysed mycotoxins above limit of detection. Concentrations are given as $\mu g \cdot kg^{-1}$ while a + indicates detection of a qualitatively determined mycotoxin. The sample information includes year of harvest, fresh (whole-crop maize) or silage (ensiled maize) sample type and the association between fungi and specific metabolites.

Sample Info			*Alternaria.* Metabolites			*Penicilium.* Metabolites							*Fusarium.* Metabolites			
**Harvest Year**	**Type**	**No**	**ALS**	**AME**	**AOH**	**AND A**	**CICO**	**MAC A**	**MAC B**	**MPA**	**ROQ A**	**ROQ C**	**DON**	**ENN B**	**NIV**	**ZEA**
2009	silage	3												47		
2009	silage	7		7									1247	47	225	
2009	silage	9		9	12								2974	85	758	209
2009	silage	12												25		
2009	silage	13														13
2009	silage	15														38
2009	silage	17												44		
2009	silage	22												36	293	32
2009	silage	23				27		+								19
2009	silage	24												29		125
2009	silage	25														130
2009	silage	27				521	+	+	+	34	+	158				
2009	silage	28				276		+			+				210	
2009	silage	29				139					+				309	64
2009	silage	31				344	+	+			+					
2009	silage	32				17										
2009	silage	33												29		
2009	silage	37												29		87
2009	silage	42												65		22
2009	silage	45				8		+								
2009	silage	46														113
2009	silage	49													211	
2009	silage	50														26
2009	silage	52													129	
2009	silage	53														9
2009	silage	54													185	
2009	silage	56													122	

Table S1. *Cont.*

Sample Info			*Alternaria.* Metabolites			*Penicilium.* Metabolites							*Fusarium.* Metabolites			
**Harvest Year**	**Type**	**No**	**ALS**	**AME**	**AOH**	**AND A**	**CICO**	**MAC A**	**MAC B**	**MPA**	**ROQ A**	**ROQ C**	**DON**	**ENN B**	**NIV**	**ZEA**
2009	silage	57												49	324	24
2009	silage	60				80					+					
2009	silage	61												38		
2007	silage	63											1777	70	165	55
2007	silage	64				136	+				+	189				
2007	silage	65				11										
2007	silage	66											1257			38
2007	silage	67				691	+	+			+					
2007	silage	69				15	+							54		45
2007	silage	70												25		
2007	silage	71				56					+			35		
2007	silage	72														10
2007	silage	74												59		9
2007	silage	75											892	86		84
2007	silage	77			24	187	+			52				63		311
2007	silage	78														31
2007	silage	80												152		17
2007	silage	82				21	+				+					
2007	fresh	83					+						2076		351	10
2007	fresh	84												33	254	19
2007	fresh	85												28	179	
2007	fresh	87												32		
2007	fresh	88														12
2007	fresh	89												129		

Table S1. *Cont.*

**Sample Info**			***Alternaria.* Metabolites**			***Penicilium.* Metabolites**							***Fusarium.* Metabolites**			
**Harvest Year**	**Type**	**No**	**ALS**	**AME**	**AOH**	**AND A**	**CICO**	**MAC A**	**MAC B**	**MPA**	**ROQ A**	**ROQ C**	**DON**	**ENN B**	**NIV**	**ZEA**
2007	fresh	90												168		
2007	fresh	91												114		19
2007	fresh	92												365	168	
2008	fresh	93														90
2008	fresh	94														29
2008	fresh	95														18
2008	fresh	96														16
2008	fresh	97														11
2008	fresh	98														25
2008	fresh	99		11									2662	159	325	666
